# A Pulse Signal Preprocessing Method Based on the Chauvenet Criterion

**DOI:** 10.1155/2019/2067196

**Published:** 2019-12-30

**Authors:** Weiguang Ni, Jianzhuo Qi, Lijia Liu, Suyi Li

**Affiliations:** ^1^College of Instrumentation and Electrical Engineering, Jilin University, Changchun, China; ^2^Physical Education College of Jilin University, Changchun, China; ^3^Xining No. 1 People's Hospital, Xining, China

## Abstract

Pulse signals are widely used to evaluate the status of the human cardiovascular, respiratory, and circulatory systems. In the process of being collected, the signals are usually interfered by some factors, such as the spike noise and the poor-sensor-contact noise, which have severely affected the accuracy of the subsequent detection models. In recent years, some methods have been applied to processing the above noisy signals, such as dynamic time warping, empirical mode decomposition, autocorrelation, and cross-correlation. Effective as they are, those methods are complex and difficult to implement. It is also found that the noisy signals are tightly related to gross errors. The Chauvenet criterion, one of the gross error discrimination criterions, is highly efficient and widely applicable for being without the complex calculations like decomposition and reconstruction. Therefore, in this study, based on the Chauvenet criterion, a new pulse signal preprocessing method is proposed, in which adaptive thresholds are designed, respectively, to discriminate the abnormal signals caused by spike noise and poor-sensor-contact noise. 81 hours of pulse signals (with a sleep apnea annotated every 30 seconds and 9,720 segments in total) from the MIT-BIH Polysomnographic Database are used in the study, including 35 minutes of poor-sensor-contact noises and 25 minutes of spike noises. The proposed method was used to preprocess the pulse signals, in which 9,684 segments out of a total of 9,720 were correctly discriminated, and the accuracy of the method reached 99.63%. To quantitatively evaluate the noise removal effect, a simulation experiment is conducted to compare the Jaccard Similarity Coefficient (JSC) calculated before and after the noise removal, respectively, and the results show that the preprocessed signal obtains higher JSC, closer to the reference signal, which indicates that the proposed method can effectively improve the signal quality. In order to evaluate the method, three back-propagation (BP) sleep apnea detection models with the same network structure and parameters were established, respectively. Through comparing the recognition rate and the prediction rate of the models, higher rates were obtained by using the proposed method. To prove the efficiency, the comparison experiment between the proposed Chauvenet-based method and a Romanovsky-based method was conducted, and the execution time of the proposed method is much shorter than that of the Romanovsky method. The results suggest that the superiority in execution time of the Chauvenet-based method becomes more significant as the date size increases.

## 1. Introduction

Within each heart beat cycle, the blood vessel presents pulsatile changes in accordance with the systolic and diastolic functions of the heart, which are termed as the pulse signals [1]. There are plenty of physiological information in pulse signals, by which some physiological parameters, such as pulse rate, blood oxygen saturation, and microcirculation, can be calculated directly or indirectly, and which can also be applied to related detection models for the evaluation of the cardiovascular, respiratory, and circulatory system statuses [2–4]. However, the pulse signals are relatively weak and can be inevitably interfered by various factors in the process of collection, especially the noises caused by the poor sensor contact and the unstable switch power, which may affect the accuracy of the subsequent related detection models [5, 6].

Generally, the noise in the signal needs to be identified by the corresponding preprocessing methods, which can then be applied to the evaluation of signal quality or the improvement of signal quality (including the suppression or removal of noise). We can use some existing preprocessing methods to improve the signal quality, especially for the above low-quality pulse signals. Li and Clifford [7] proposed a PPG signal preprocessing algorithm based on dynamic time warping (DTW), which evaluated the signal quality by means of analyzing the characteristics related to signal quality through a multilayer perception neural network. Karlen et al. [8] developed a preprocessing algorithm that can estimate the quality of PPG signals in real time. By using the cross-correlation algorithm for segmented PPG signals, the signal quality index (SQI) was obtained and the poor-quality data were removed by the index. Kou [9] defined the dynamic variable coefficient by using the mean value and variance, and the thresholds were determined by window sliding and iterative calculation to detect the poor-sensor-contact noises, which not only evaluated the signal but also removed the outliers. Li et al. [10] proposed a joint algorithm which combined the time domain and the frequency domain to evaluate the pulse signals. And, the fundamental waves in the frequency domain were analyzed by means of the quality factors used in physics and engineering, and in combination with the valid single edge counts in the time domain, the low-quality signals were selected and removed. Koneshloo and Du [11] proposed a PPG signal preprocessing method on a joint basis of pursuit linear program. By reconstructing and analyzing the sequence correlation of PPG signals, the adaptive removal of noise was achieved, which provided high-quality pulse signal for the subsequent processing. Li et al. [12] proposed a simple real-time denoising method based on double median filter to preprocess the PPG signal, which improved the quality of signals by effectively suppressing the noise and preserving the essential morphological features from PPG signals. Wang et al. [13] applied empirical mode decomposition (EMD) to the processing of the dynamic pulse data. They selected the specific components to reconstruct the signals and extracted important features in the original signals by applying multiscale filter and accumulated energy contribution rate filter to the components that were obtained from decomposition, resolving the problem of breaks in the dynamic pulse signals, namely, the problem of poor-sensor-contact. Sun et al. [14] firstly filtered the original pulse signals, then extracted the wave peak characteristics of the filtered signals, and lastly selected the signals of good quality by applying variance discrimination to the characteristics, which improved the computation accuracy of physiological parameters. The application of the above preprocessing methods has made a great contribution to the evaluation or improvement of pulse signal quality. However, the methods applied for removing the spike noise and the poor-sensor-contact noise are so complex that they have to use multiple iterative calculations, decompositions, reconstructions, and so on and thus occupy many system resources, not favorable for the subsequent related physiological parameters and the establishment of detection models. Therefore, it is of great significance to adopt a simpler and more effective preprocessing method for the removal of these noises.

In view of the fact that the abnormal conditions, such as poor-sensor-contact and instrument malfunctions, are small probability events in the process of signal collection, and theoretically, the gross errors are the small probability errors that exceed the normal error range in specified conditions. Therefore, the noises collected in these abnormal conditions can be analyzed and processed according to the discrimination criterion of gross error, being identified and then removed. Due to the complexity of the existing algorithms and referring to the discrimination criterion of the gross error and its applicable conditions, in the design of the preprocessing method combined with the characteristics of the actual abnormal noises, the widely applicable Chauvenet criterion that does not contain multiple iterations is selected as the basic principle.

To sum up, this paper has proposed a new pulse signal preprocessing method based on the Chauvenet criterion, which is highly efficient in implementation, and is used to discriminate the noises occurring in the conditions of poor-sensor-contact and unstable switch power. According to the gross error discrimination criterion and the characteristics of the noises, adaptive thresholds are designed to discriminate the spike noise and the poor-sensor-contact noise and then the pulse signals in the MIT-BIH database are used to validate the effectiveness.

## 2. Data

**Figure 1:**
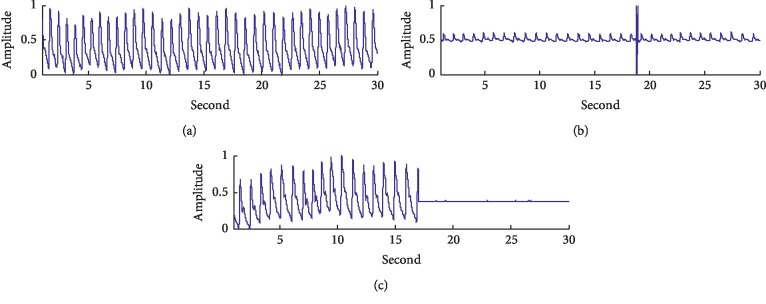
Pulse signals: (a) high-quality signal; (b) signal with spike noise; (c) signal with poor-sensor-contact noise.

The pulse signals used in this research, taken from the MIT-BIH Polysomnographic Database [15, 16] (https://www.physionet.org/content/slpdb/1.0.0/), were obtained from the detection of 16 subjects in the sleeping lab of Boston's Beth Israel Hospital. All the subjects were male, aged 32 to 56 (43 on average), and weighed 89 to 152 kg (119 kg on average). The data include 81 hours' pulse signals with a sampling frequency of 250 Hz and corresponding sleep apnea syndrome annotations, among which a segment of valid signals of high quality is shown in Figure 1(a). Apart from the baseline drift, the power frequency noise and the electromyography interference, pulse signals collected in the actual conditions, may also include spike noises caused by unstable switch power and noises caused by poor sensor contact. As is shown in Figure 1(b), the amplitude of the spike noise is very high, almost reaching the maximum of the AD converter. The noisy signal occurring in the condition of poor sensor contact is shown in Figure 1(c), with no signal input in the second half segment.

Noisy signals of low quality are not suitable for the subsequent detection model. Therefore, an effective algorithm needs to be designed to discriminate such signals. There are 81 hours of pulse signals in the MIT pulse database, which is mainly used for the detection of sleep apnea and annotations are already given by experts to the signals of 30 seconds in each segment, with 9,720 segments in total. As is known, the subsequent analysis is based on the quality of the data, so the noises in the pulse signals affect the accuracy in the establishment of the sleep apnea model. Therefore in this study, in a similar pattern, quality annotations of the data are given to the signals of 30 seconds in each segment, which is used for the study of noise discrimination algorithm. It is discovered that there are 35 minutes' poor-sensor-contact noises and 25 minute' spike noises in all the 81 hours of pulse signals.

## 3. Method

### 3.1. Characteristics and Basic Principles of the Chauvenet Criterion

This study has proposed a discrimination method for noisy pulse signals based on the criterion of the gross error discrimination. The widely used criterions are the Pauta criterion, the Romanovsky criterion, the Dixon criterion, the Grubbs criterion, and the Chauvenet criterion [14]. The Pauta criterion is applicable only in the precondition of sufficient times of collection, so it cannot be applied to a small amount of signals. In the Romanovsky criterion, iterative calculations are needed to determine whether there are gross errors in each segment of signals. With this method, noises cannot be detected from a great amount of data at once. In both the Grubbs criterion and the Dixon criterion, data need to be sorted firstly and only the first or last segment of signals can be detected by each calculation. Multiple iterative calculations need to be conducted, which is a complex and inefficient process. Neither a multiple iteration nor the sorting of data is necessary for the Chauvenet criterion, which is not limited by the amount of data so it is easier to conduct quick and accurate discrimination.

The Chauvenet criterion is a strict gross error discrimination criterion based on the equal confidence probability [17]. A probability range, including all the samples in the data set and centered on a mean value, is determined, and all the data outside of the range are taken as abnormal and to be removed from the data set. If the number of measurements recorded is *n*, also called sample size, and then the confidence probability is 1 − (1/2*n*). The quantity (1/2*n*) corresponds to the combined probability represented by the two tails of the normal distribution, and due to its symmetry, it is possible to consider only the probability (1/4*n*) of one tail. The Chauvenet coefficient, which is also referred to as the maximum allowable deviation, can be achieved by finding the *z*-score corresponding to the (1/4*n*) portion of the confidence probability, so it is only related to the sample size *n*. In the condition of normal distribution (the average value of the distribution is 0 and the standard deviation is 1), the Chauvenet coefficient can be calculated by the inverse function value based on 4*n* or an empirical formula 1+0.4 ln(*n*). If the absolute value of the difference between a detected value and the mean value is greater than the product of the standard deviation and the Chauvenet coefficient, the detected value is determined as containing gross errors.

### 3.2. Pulse Signal Preprocessing Method with the Adaptive Thresholds Based on the Chauvenet Criterion

In this research, the abnormal pulse signals are discriminated by the Chauvenet criterion, and the steps are as follows.

#### 3.2.1. Calculation of Characteristic Samples

For any set of the original data including the pulse signals in the *m*-section with a length of *n* for each section, $X=\left(\begin{array}{ccc} x_{11} & \cdots & x_{1n}\\ \vdots & \ddots & \vdots \\ x_{m1} & \cdots & x_{mn} \end{array}\right)$. In this application, the pulse signals last 30 seconds in each segment, with 9,720 segments in total, and thus *n* equals 7,500 and *m* equals 9,720.

*(1) The Characteristic Samples of Spike Noise*. According to the characteristics of the spike noise amplitude, the original segmented signals are taken the subject for detection. Therefore, firstly, the mean value for each original data segment is calculated in turn to form the mean value sample, mean_*i*_=∑_*j*=1_^*n*^*x*_*ij*_/*n*, *i*=1,2,…, *m*; and next, the standard deviation for each data segment is calculated in turn to form the standard deviation sample, ${\text{std}}_{i}=\sqrt{\sum_{j=1}^{n}{\left(x_{ij}-{\text{mean}}_{i}\right)}^{2}/\left(n-1\right)}$, *i*=1,2,…, *m*; In this application, *n* equals 7,500, *m* equals 9,720, and std_*i*_ is a 9,720 dimensional array obtained by calculation.

*(2) The Characteristic Samples of Poor-Sensor-Contact Noise*. According to the features of poor-sensor-contact noises, we divide the 9,720 segments of pulse signals into 10 groups and calculate the mean value of the standard deviation samples of each group, mean std=∑_*i*=1_^*m*^std_*i*_/*m*, and then calculate the standard deviation of the standard deviation samples, $\text{std std}=\sqrt{\sum_{i=1}^{m}{\left({\text{std}}_{i}-\text{mean std}\right)}^{2}/\left(m-1\right)}$.

#### 3.2.2. Determination of the Adaptive Thresholds

Adaptive thresholds are designed to conduct more accurate discrimination. For the subject *X* to be measured, with a quantity of *t*, the equation of the adaptive threshold *T* is as follows:

(1) $$\begin{array}{c} T=W_{t}S_{X}=\left\{1+0.4\;\ln\left(t\right)\right\}\sqrt{\frac{\sum_{i=1}^{t}{\left(X_{i}-\bar{X}\right)}^{2}}{t-1}}, \end{array}$$

where $\bar{X}$ is the sample mean value, *S*_*X*_ is the sample standard deviation, and *W*_*t*_ is the Chauvenet coefficient calculated by the empirical formulae in this application.

*(1) The Threshold Design for Spike Noise Discrimination*. Applying equation (1) to design the threshold for the spike noise discrimination, the original segmented signals *X*_*ij*_ are selected as detection subjects, *i*=1,2,…, *m*; *j*=1,2,…, *n*; the quantity of detection subjects is determined by the sample size of each segment of signals *t*=*n*, and the spike noise threshold *T*_*pn*_ is

(2) $$\begin{array}{c} T_{pn}=W_{n}{\text{std}}_{i}=\left\{1+0.4\;\ln\left(n\right)\right\}\sqrt{\frac{\sum_{j=1}^{n}{\left(X_{ij}-{\text{mean}}_{i}\right)}^{2}}{n-1}}, \end{array}$$

where *n* stands for the sample size and *W*_*n*_ is the Chauvenet coefficient for spike noise discrimination. In this application, *n* equals 7,500, *W*_*n*_=1+0.4 ln(*n*)=1+0.4 ln(7500)=4.569, std_*i*_ is calculated by Step 1, and then we can obtain *T*_*pn*_.

*(2) The Threshold Design for Poor-Sensor-Contact Noise Discrimination*. For the poor-sensor-contact signals, the standard deviation sample std_*i*_ is selected as detection subjects, *i*=1,2,…, *m*; the quantity of detection subjects is determined by the number of signal segments *t*=*m*, and the threshold of poor-sensor-contact noise *T*_*pc*_ is

(3) $$\begin{array}{c} T_{pc}=W_{m}\text{std std}=\left\{1+0.4\;\ln\left(m\right)\right\}\sqrt{\frac{\sum_{i=1}^{m}{\left({\text{std}}_{i}-\text{mean std}\right)}^{2}}{m-1}}, \end{array}$$

where *m* stands for the segments and *W*_*m*_ is the Chauvenet coefficient for poor-sensor-contact noise discrimination. In this application, *m* equals 972, *W*_*m*_=1+0.4 ln(*m*)=1+0.4 ln(972)=3.752, std std is calculated by Step 1, and then we can obtain *T*_*pc*_.

#### 3.2.3. Discrimination of Spike Noise and Poor-Sensor-Contact Noise

*(1) Spike Noise Discrimination*. As for the abnormal signal with spike noise, since the amplitude of the spike noise is extremely large, the Chauvenet criterion is used for each original segmented signal to determine whether there is an abnormal value. If it does, the segmented signal is taken as a spike noise. In other words, if the absolute value of the difference between a certain detected value and the mean value of signals in the segment is greater than the spike noise threshold, as is shown in equation (4), the segmented signal being detected is taken as an abnormal signal including spike noise. All the signal segments are discriminated in turn, and the locations of the discriminated spike noises are recorded in the set P1:

(4) $$\begin{array}{c} \left|x_{ij}-{\text{mean}}_{i}\right|>T_{pn}. \end{array}$$

*(2) Poor-Sensor-Contact Noise Discrimination*. As for the poor-sensor-contact noises, due to the existence of sudden amplitude changes in pulse signal when sensor is poorly contacted, the Chauvenet criterion is used for the standard deviation sample to discriminate if there is any abnormal value among them. If it does, the segment of signals corresponding to this abnormal standard deviation is taken as poor-sensor-contact noises. In other words, if the absolute value of the difference between a standard deviation and the mean value of it is greater than the threshold of the poor-sensor-contact, as is shown in equation (5), the signal corresponding to this standard deviation is taken as a poor-sensor-contact noise. The locations of the discriminated poor-sensor-contact noises are recorded in the set P2:

(5) $$\begin{array}{c} \left|{\text{std}}_{i}-\text{mean std}\right|>T_{pc}. \end{array}$$

#### 3.2.4. Noise Removal

The noises are removed from the original signals to obtain the final preprocessed signals according to the location of the abnormal signals, which is the union of the set P1 and the set P2.

## 4. Results and Discussion

**Figure 2:**
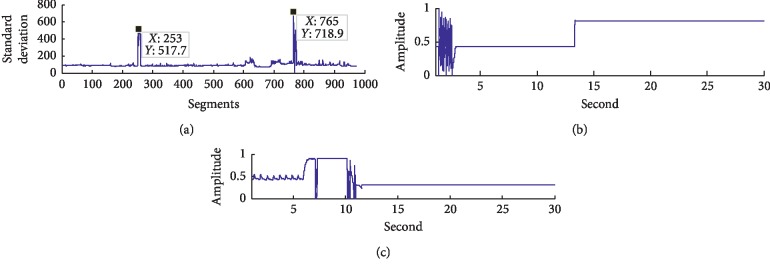
The detection results of the signals with poor-sensor-contact noises: (a) the standard deviation of group 1; (b) the pulse signal in segment 253; (c) the pulse signal in segment 765.

The 81 hours' original signals in the data base were divided into 9,720 segments in time sequence, with 30 sec for each segment. Then, all the 9,720 segments were evenly divided into 10 groups, with 972 segments in each. The proposed method in this study was applied to each group. For group 1, the detection results are shown in Figure 2 and the standard deviations of the 972 segments are shown in Figure 2(a). The abscissa represents the locations of the segmented signals of group 1, with 972 segments in total. The ordinate represents the standard deviations corresponding to the segmented signals.

As can be seen in Figure 2(a), the standard deviations of the signals in segment 253 and segment 765 are much higher than those of other signals, so it can be determined that the two segments include poor-sensor-contact noises. The waveforms of the signals in segment 253 and segment 765 are shown in Figures 2(b) and 2(c), and in order to clearly compare the waveforms of the signals, their amplitudes are normalized from 0 to 1. The abscissa stands for the time of data collection and the ordinate for the normalized amplitudes. It can be seen that there are poor-sensor-contact noises in the signals plotted in Figures 2(b) and 2(c).

**Figure 3:**
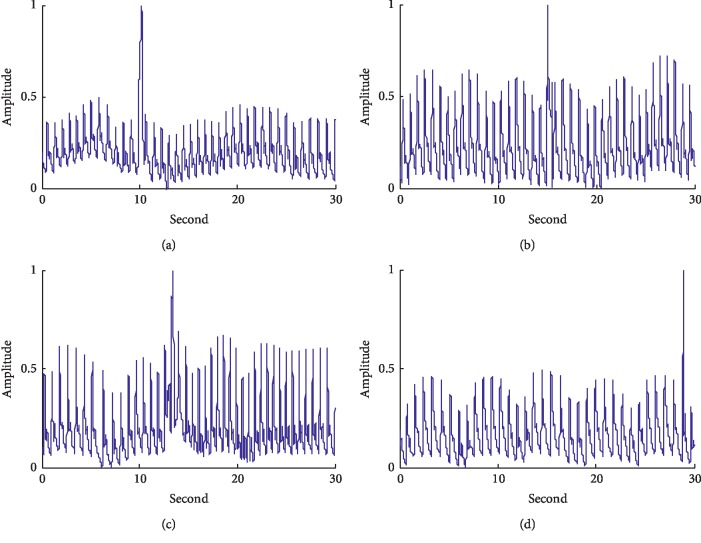
The detection results of the signals with spike noises: (a) the pulse signal in segment 13: (b) the pulse signal in segment 201: (c) the pulse signal in segment 331: (d) the pulse signal in segment 719.

The proposed method in this study was applied to group 7, and the spike noises detected are shown in Figures 3(a)–3(d), corresponding to segments 13, 201, 331, and 719, respectively. It can be seen that there are spike noises with abnormal amplitudes in all of the four segments.

The proposed method was applied to 10 groups of pulse signal, respectively, and the two types of noises (spike noise and poor-sensor-contact noise) were detected in each group. The discrimination results are shown in Table 1. The accuracy of the proposed method, *Accuracy_D*, is calculated as

(6) $$\begin{array}{c} Accuracy\_D=\frac{\text{TP}+\text{TN}}{\text{TP}+\text{TN}+\text{FP}+\text{FN}}\times 100\%, \end{array}$$

where TP is true positive when noises discriminated as noises, TN is true negative when normal signals discriminated as normal signals, FP is false positive when normal signals discriminated as noises, and FN is false negative when noises discriminated as normal signals.

As is seen in Table 1, the discrimination accuracy of each group of signals is over 99%. In the results, 9,684 segments out of a total of 9,720 were correctly discriminated, and the average accuracy of the proposed method in discrimination has reached 99.63% with the average relative error as 0.37%.

To evaluate the noise removal effect by the proposed method, a simulation experiment is conducted to compare the similarity of the two signals by calculating the Jaccard Similarity Coefficient (JSC) (shown in equation (7)). The closer the JSC value is to 1, the higher the similarity between the two signals:

(7) $$\begin{array}{c} \text{JCR}=\frac{\left|f\left(n\right)\cap \hat{f}\left(n\right)\right|}{\left|f\left(n\right)\cup \hat{f}\left(n\right)\right|}, \end{array}$$

where *f*(*n*) represents the reference signal and $\hat{f}\left(n\right)$ represents the noisy signal or processed signal.

**Figure 4:**
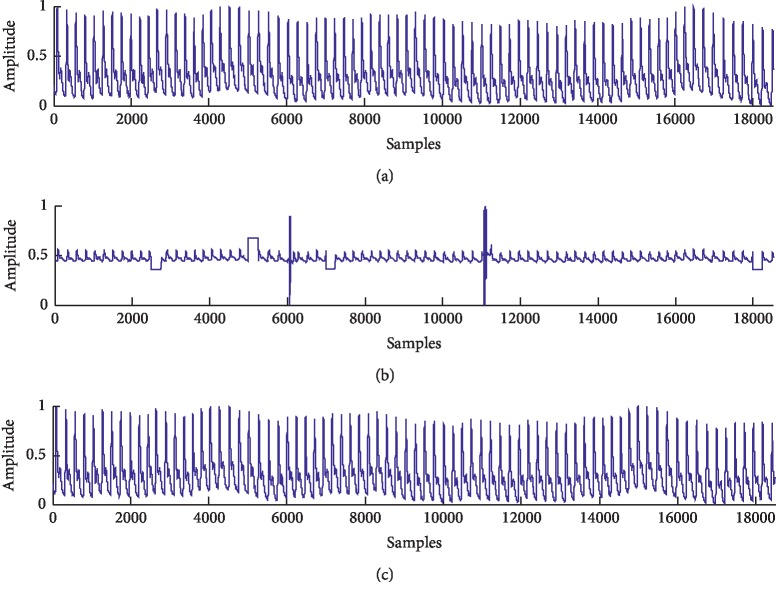
The pulse signals in simulation experiment: (a) *Rsig*; (b) *Nsig*; (c) *Psig*.

A fraction of good-quality pulse signals serving as the reference signal (named as *Rsig*) (shown in Figure 4(a)), a noisy signal (named as *Nsig*) (shown in Figure 4(b)) is synthesized by adding the spike noise and the poor-sensor-contact noise to the reference signal. And then, the preprocessed signal (named as *Psig*) (shown in Figure 4(c)) is obtained through the proposed method.

Comparing the waveforms from Figure 4, the spike noise and the poor-sensor-contact noise are greatly removed. Besides the visual comparison, JSC is used to evaluate the proposed method quantitatively. The JSC calculated between *Rsig* and *Nsig* is 0.77 and that between *Rsig* and *Psig* is 0.93. The result shows that our proposed method obtains higher JSC, closer to the reference signal, which suggests the quality of the signal is improved.

In order to validate the reliability of the method, a detection model of sleep apnea based on back-propagation (BP) neural network was set up, with the original pulse signals, the denoised signals were obtained by a median filtering method [12], and the preprocessed signals by using the proposed method were used as the model input, respectively, and the sleep apnea syndrome annotations as model output, which were marked as the models ORIP-Apnea, Denoising-Apnea, and PREP-Apnea, respectively. Before preprocessing, there were 9,720 segments of pulse signal, and 9,606 segments were left after removing noises with the proposed method. The detection model is a three-layer BP neural network, with the number of neuron in the hidden layer being 50. The transfer function in the hidden layer is *sigmoid*, the transfer function in the output layer is *softmax*, the performance function is *cross-entropy*, and the training function is *trainscg*. The calculation for the accuracy of the model, *Accuracy_M*, is shown as

(8) $$\begin{array}{c} Accuracy\_M=\frac{\text{TPA}+\text{TNA}}{\text{TPA}+\text{TNA}+\text{FPA}+\text{FNA}}\times 100\%, \end{array}$$

where TPA stands for true sleep apnea, TNA for true nonsleep apnea, FPA for false sleep apnea, and FNA for false nonsleep apnea.

10-fold cross-validation was used to calculate the recognition rate (RR, the accuracy calculated using the training data set) and prediction rate (PR, the accuracy calculated using the test data set) of ORIP-Apnea, Denoising–Apnea, and PREP-Apnea, as is shown in Table 2.

As is shown in Table 2, the average recognition rate and prediction rate of ORIP-Apnea are 78.11% and 77.61%, respectively, and those of Denoising-Apnea and PREP-Apnea are 79.12%, 79.06%, 81.96%, and 81.06%, respectively. Both the recognition rate and prediction rate of PREP-Apnea are higher than those of ORIP-Apnea and Denoising-Apnea, with the recognition rate increased by 3.85% and the prediction rate increased by 3.45% after preprocessing the original signal, which is mainly because the proposed preprocessing method has identified and processed noises that are always mistaken for apnea signals. The preprocessed signals can improve the accuracy when applied to the apnea detection model, which indicates that the proposed method is a reliable preprocessing method for pulse signals.

In order to prove the efficiency, we conducted the experiment for execution time comparison by using a computer (64-bit based PC configuration: Windows 7 64 bit, Matlab R2014a 64 bit, Intel Core i5-7500, 3.4 GHz, 32 GB RAM) and compared the execution time of the proposed Chauvenet-based method with that of a Romanovsky-based method which needs multiple iterative calculations. The comparisons of the execution time between the two methods are shown in Table 3.

If a record contains *M* sampling points, the mean value and standard deviation will be calculated *M* times by the Romanovsky-based method, so with *N* records, the calculations will be *N* × *M* times. However, the mean value and standard deviation will be calculated once per record by the Chauvenet-based method, so with *N* records, the calculations will be *N* times only. As is shown in Table 3, we can see that the RET increases greatly with the number of records *N* going up. The performance indicates that CET is shorter than RET, and the superiority of the proposed method in execution time is more significant as the size of data increases.

## 5. Conclusion

A preprocessing method based on the Chauvenet criterion is proposed to solve the problem that noises severely affect the accuracy of the subsequent detection models. Referring to the error theory, adaptive thresholds for noises discrimination are designed according to the characteristics of the spike noise and the poor-sensor-contact noise and those of the original pulse signals. The noises are removed by means of discrimination based on the Chauvenet criterion, so that the signal quality is improved. The pulse signals from the MIT-BIH database are preprocessed with the proposed method, and it is found that the discrimination accuracy has reached 99.63%. The noise removal effect is evaluated through the similarity comparison experiment. JSCs, which are calculated before and after the noise removal respectively, have increased from 0.77 to 0.93. The results show that the quality of the signal is improved through the proposed method. For validating the reliability of the method, sleep apnea detection models, ORIP-Apnea, Denoising-Apnea, and PREP-Apnea, based on the BP neural network are set up with the original pulse signals, denoised signals, and preprocessed signals as the input, respectively. Both the recognition rate and prediction rate of PREP-Apnea are higher than those of ORIP-Apnea and Denoising-Apnea. Compared with those of ORIP-Apnea, the recognition rate and the prediction rate of PREP-Apnea have increased by 3.85% and 3.45%, respectively. In addition, the comparison experiment of execution time is conducted to prove the efficiency, and the execution time of the proposed method is much shorter than that of the Romanovsky method. The performance shows that, in processing speed, the superiority of the proposed method is more significant as the size of the data increases. The above results indicate that the proposed method can effectively improve signal quality and the detection accuracy, which has a potential significance for the detection of related diseases with pulse signals.

## Figures and Tables

**Table 1:** The discrimination results of the proposed method.

No.	True	False	TP	TN	FP	FN	Accuracy (%)	Error (%)
1	968	4	18	950	2	2	99.59	0.41
2	972	0	8	964	0	0	100.00	0.00
3	968	4	8	960	2	2	99.59	0.41
4	969	3	14	955	2	1	99.69	0.31
5	970	2	2	968	0	2	99.79	0.21
6	969	3	12	957	0	3	99.69	0.31
7	968	4	4	964	3	1	99.59	0.41
8	969	3	17	952	0	3	99.69	0.31
9	966	6	11	955	0	6	99.38	0.62
10	965	7	5	960	6	1	99.28	0.72
Total	9684	36	99	9585	15	21	—	—
Average	—	—	—	—	—	—	99.63	0.37

**Table 2:** Comparisons of recognition rate and prediction rate among ORIP-Apnea, Denoising–Apnea, and PREP-Apnea.

No.	ORIP-Apnea		Denoising-Apnea		PREP-Apnea	
	RR (%)	PR (%)	RR (%)	PR (%)	RR (%)	PR (%)
1	78.41	78.02	79.12	79.04	81.74	80.92
2	78.64	78.80	78.68	79.09	82.51	81.51
3	77.13	77.04	78.76	79.02	82.37	81.07
4	78.16	78.55	79.84	79.44	80.82	80.91
5	77.28	76.11	79.64	79.12	81.67	80.87
6	77.62	78.42	78.58	78.86	82.02	81.33
7	79.33	76.16	79.23	79.17	81.33	81.58
8	78.50	78.22	78.65	78.83	82.56	80.04
9	77.82	77.92	79.26	79.01	83.55	82.02
10	78.21	76.83	79.43	79.06	81.03	80.37
Average	78.11	77.61	79.12	79.06	81.96	81.06

**Table 3:** Comparisons of the execution time between the proposed Chauvenet-based method and the Romanovsky-based method.

No.	*N*=10		*N*=100		*N*=1000	
	CET (s)	RET (s)	CET (s)	RET (s)	CET (s)	RET (s)
1	1.54	12.73	1.56	111.98	1.75	1040.93
2	1.55	12.56	1.56	113.40	1.75	1041.74
3	1.54	12.59	1.56	112.80	1.73	1022.54
4	1.56	12.54	1.56	109.51	1.79	1033.36
5	1.54	12.60	1.56	113.33	1.75	1023.35
6	1.55	12.58	1.56	112.52	1.75	1035.48
7	1.54	12.60	1.56	111.50	1.73	1029.24
8	1.54	12.55	1.57	111.98	1.74	1034.65
9	1.54	12.58	1.56	112.08	1.76	1037.84
10	1.55	12.66	1.57	112.13	1.74	1025.65
Average	1.54	12.60	1.56	112.12	1.75	1032.48

*Note*. *N* stands for the segments of pulse signals processed by the two methods and CET and RET represent the execution time using the proposed Chauvenet-based method and the Romanovsky-based method, respectively.

## Data Availability

The pulse signals used in this research are taken from the MIT-BIH Polysomnographic Database (https://www.physionet.org/content/slpdb/1.0.0/).
